# Autonomic measures identify stress, pain, and instability associated with retinopathy of prematurity ophthalmologic examinations

**DOI:** 10.3389/fpain.2022.1032513

**Published:** 2022-11-22

**Authors:** Vivian Onuagu, Fumiyuki Gardner, Ajay Soni, Kim K. Doheny

**Affiliations:** ^1^ Department of Neonatology, Mountain View Hospital Las Vegas, Las Vegas, NV, United States; ^2^ Department of Pediatrics, Penn State College of Medicine, Hershey, PA, United States; ^3^ Department of Ophthalmology, Penn State College of Medicine, Hershey, PA, United States; ^4^ Department of Neural and Behavioral Sciences, Penn State College of Medicine, Hershey, PA, United States

**Keywords:** retinopathy of prematurity (ROP), high frequency heart rate variability (HF-HRV), skin conductance (SC), neonatal pain, neonatal stress, preterm infants

## Abstract

**Background:**

Retinopathy of prematurity (ROP) ophthalmologic examinations cause stress and pain. Infants’ stress and pain can be measured non-invasively using skin conductance (SC) and high frequency heart rate variability (HF-HRV), reflecting sympathetic-mediated sweating and parasympathetic activity, respectively.

**Objectives:**

To test the utility of SC to detect sympathetic activation during ROP examination, and the contribution of HF-HRV to predict stability post-examination.

**Methods:**

In this prospective, single center study, we measured SC continuously pre-, during, and post-examination, and HRV at 24 h pre-ROP examination. Clinical data included stability [apneas, bradycardias, and desaturations (A/B/Ds)], and interventions post-examination.

**Results:**

SC increased 56% above baseline during ROP examination (*p* = 0.001) and remained elevated post-examination (*p* = 0.02). *Post-hoc* analysis showed higher illness acuity, represented by need for respiratory support, was associated with lower HF-HRV at 24 h pre-ROP examination (*p* = 0.001). Linear regression indicated lower HF-HRV at 24 h pre-examination contributed to the need for higher intervention (i.e., stimulation to breathe, oxygen support) particularly among infants with higher illness acuity [*F*(1, 15) = 5.05, *p* = 0.04; *β* = −1.33, *p* = 0.04].

**Conclusion:**

ROP examination induced a 2-fold increase in sympathetic activation which remained above baseline in recovery. Also, we propose that the low parasympathetic tone associated with autonomic imbalance contributes to instability and need for higher intervention to assure stabilization with A/B/D events. Our findings provide insight into the underestimation of adverse events associated with ROP examination and identification of infants who may be more vulnerable to potential sequelae following ROP examinations.

## Introduction

Retinopathy of prematurity (ROP) is the leading cause of preventable childhood blindness in developed countries and ophthalmologic examinations necessary for diagnosis of the disorder have been shown to cause stress and pain (1, 2). The American Academy of Pediatrics’ recommendation for ROP screening includes infants born at gestational age (GA) ≤30 weeks or birthweight (BW) ≤1,500 g, or GA >30 weeks or BW 1,500–2,000 g with unstable course including cardiorespiratory support or deemed as high risk for ROP by the pediatrician or neonatologist (3). These ROP screenings are part of the standard of care amongst other painful yet necessary procedures in hospitalized preterm infants. Such noxious events which trigger a pain response in newborns (cry, elevated heart rate, facial grimace) occur with notable frequency in the neonatal intensive care unit (NICU) and raise concerns related to the establishment of the protective, nurturing environment necessary enhance growth and development for the particularly vulnerable preterm population (4–6). Moreover, repeated pain-inducing procedures in preterm infants have been associated with stress dysregulation and contribute to adverse short and long-term neurodevelopmental sequelae (6–8).

Pain assessment in preterm infants can be challenging due to non-discriminating facial actions and less robust vocalizations as compared to term infants (9–11). There are numerous infant clinical pain scales that utilize the combination of behavioral and physiological responses; however, there is no single gold-standard, and inadequate training in scale use may lead to under-recognition and under-treatment of pain in preterm infants (9–12).

Skin conductance (SC) provides valuable information as a sensitive and specific adjuvant to clinical pain scales as its utility has been well demonstrated to assess sympathetic activation associated with noxious stimuli (i.e., heel stick, endotracheal intubation, post-operative pain) in infants (7, 13–18). Less is known, however, regarding the extent to which autonomic measures such as SC would be useful during the ROP examination to measure sympathetic activation in real time. When the sympathetic branch of the autonomic nervous system is activated (during pain, stress, fear), sympathetic nerve fibers release acetylcholine, which acts on muscarinic receptors with subsequent release of sweat from eccrine sweat glands, known as sympathetic-mediated sweating (14). When sweat reaches the skin surface, skin conductance increases; hence surface electrodes detect conductance changes recorded as the number of waves per second depicting the frequency of sympathetic nervous system firing, while amplitude of the waves depicts the strength of firing (14). SC also referred to as electrodermal responses (EDRs) per second and their amplitudes are averaged over time, as the mean of peaks (MPs). SC is particularly useful to assess sympathetic activation associated with painful procedures (13–17) and it is not affected by hemodynamics, cardiorespiratory changes, or cardiovascular drugs (18).

Another autonomic measure beneficial in identifying vulnerability to the effects of pain and stress is heart rate variability (HRV). HRV obtained from electrocardiogram (ECG) recordings, has been shown to portray the maturation and functional status of the autonomic nervous system (8, 19, 20). Time and frequency domains as well as nonlinear methods can be used for HRV analysis (21, 22). For the frequency domain analysis, signals are filtered to different spectral frequencies including low-frequency (LF) and high frequency (HF) bands using a fast Fourier transform of inter-beat-intervals of successive heart beats (21, 22). The high frequency spectrum (HF-HRV) represents parasympathetic outflow, i.e., cardiac vagal tone (21, 23). Furthermore, HF-HRV has been shown to be positively associated with stress resiliency and diminished neonatal morbidity (23–26). Thus, in this study we used frequency domain analysis and HF-HRV as the measure of interest to reflect resting parasympathetic tone as an indicator of stress resilience.

As described, both SC and HRV are validated for assessing procedural pain/stress and recovery in infants with studies identifying the utility of SC for objectively quantifying sympathetic arousal during neonatal painful procedures (13–18) and HF-HRV to identify resting parasympathetic tone and vulnerability to pain/stress exposures (23–26). SC measures sympathetic activation and is regulated by higher brain centers (14), while HF-HRV is regulated by the brainstem and measures parasympathetic activation (23). The study objectives were to test the utility of SC to detect sympathetic activation during the ROP examination, and to determine the contribution of parasympathetic tone reflected in HF-HRV pre-examination to predict stability post-examination.

## Materials and methods

### Patients

This cross-sectional, single-center, observational study was conducted on a cohort of preterm born infants who underwent ROP ophthalmologic examinations at a level IV NICU within an academic medical center in southcentral Pennsylvania. Eligible participants were hospitalized preterm born infants who qualified for ROP examination. Exclusion criteria included chromosomal, neurologic, or cardiac anomalies.

### Ethics

The study protocol was reviewed and approved for ethical and regulatory compliance by the Institutional Review Board (IRB) affiliated with the academic medical center. Informed written parental consent was obtained for each subject prior to study enrollment.

### Procedure

We confirmed that subjects did not have any painful procedures within 12 h of the ROP examination. Subjects were swaddled and received oral sucrose, as well as tetracaine eye drops prior to their ROP examination. The examinations were performed by one pediatric ophthalmologist and included: administration of anesthetic drops, lid retraction with an Alfonso lid speculum, scleral indentation and retinal visualization, retractor removal from the first eye and insertion into the second eye, scleral indentation and retinal visualization to lid retractor removal from the second eye. Following the ROP examination observations, the subjects received routine care.

### Measures

#### Skin conductance (SC)

Skin Conductance activity was measured using the Med-Storm™ device (Med-Storm Innovation, Oslo Norway). This device has been used previously at our institution in pain/stress studies of preterm infants as described by Zeiner, Storm, and Doheny (7). SC surface electrodes (Conmed® Corporation, Utica, NY, USA) were used to detect SC using 3 electrodes: a measuring electrode on the plantar surface of the foot, a counter current electrode on the dorsal surface of the foot, and a reference voltage electrode on the medial thigh.

Following electrode placement, activities related to the care/handling of subjects and the ROP examination were documented by time, frequency, and duration in a computerized log. SC was measured continuously from 20 min prior to the ROP examination, during the ROP examination, and through the 20 min after the examination.

**Figure 1:**
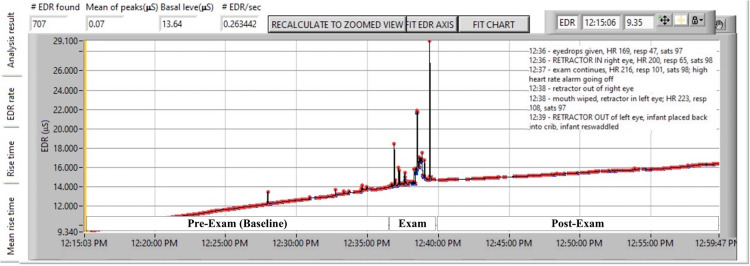
Representative subject showing continuous skin conductance before, during, and after ROP examination. ROP examination reflects noxious effects of anesthetic eye drops, lid retraction, scleral indentation and retinal visualization, retractor removal from the first eye and insertion into the second eye, scleral indentation and retinal visualization to lid retractor removal from the second eye. Each waveform *peak* (or electrodermal response, EDR) is indicated by a red marker. The *frequency of peaks* divided by the time of the epoch in seconds is EDR/sec or *peaks/sec*. The average height of peaks is represented as *mean of peaks*.

Following data acquisition, analysis was done offline using software from the manufacturer (Med-Storm™) to quantify the: number of electrodermal responses (EDRs) per second; and the average amplitude of the responses, as mean of peaks, MPs (µsiemens). See Figure 1 as a representative example.

#### Heart rate variability (HRV)

Heart rate variability (HRV) was measured using standard lead II electrocardiograph (ECG) placement and a portable data acquisition system as previously described (25). Each measurement session lasting 30–40 min was standardized for time of day (12 pm/noon—5 pm), and approximately 1 hour after care and feeding times, to control for circadian variations and to ensure that infants were calm and in a quiet, fed state. We chose the periods of 24 and 48 h proximal to the scheduled ROP examination to obtain repeated measures of HRV to determine the infant's resting parasympathetic tone.

After data acquisition, each 120 s segment of R-R wave data was manually screened offline to ensure that it was free of ectopic beats and/or movement artifacts. For each subject, on average 12–15 of 120 s segments were analyzed for high frequency (HF-HRV) spectral power, in the range of 0.3–1.3 Hz, as previously described (25) to represent the parasympathetic activity associated with faster breathing rates of our preterm sample.

#### Illness acuity and infant stability

Illness acuity at the time of the ROP examination was determined by need for respiratory support rated categorically from zero to five where 0 = room air, 1 = low flow nasal cannula, 2 = high flow nasal cannula, 3 = continuous positive airway pressure (CPAP), 4 = noninvasive intermittent mandatory ventilation (NIMV) or noninvasive neurally adjusted ventilatory assist (NAVA) and 5 = synchronized intermittent mandatory ventilation (SIMV); placing subjects into one of two respiratory support groups: positive pressure support (high flow nasal cannula >2 L/min, CPAP, NIMV or SIMV) and non-positive pressure support (low flow nasal cannula or room air).

Electronic medical records (EMR) were reviewed for the frequency and severity of apneas, bradycardias, and desaturations (A/B/D) for the 72 h post ROP-examination. A/B/Ds were defined as: apnea, pause in breathing for ≥20 s, or <20 s with bradycardia and/or desaturation; bradycardia, heart rate <100 bpm; and desaturation, SPO2 <85% for postmenstrual age (PMA) <36 weeks or SPO2 <90% for PMA ≥36 weeks.

Interventions for A/B/Ds were categorized by severity using the following scale from zero to five: 0 = no intervention; 1 = mild stimulation (e.g., gentle rub of foot or back); 2 = moderate stimulation (e.g., repositioning onto the back with a head and shoulder roll); 3 = blow by oxygen; 4 = bag, mask ventilation (BMV); and 5 = increase in the level of respiratory support in addition to BMV.

#### Demographic and descriptive information

Seventy-four hospitalized neonates were screened for eligibility between October 2018 and June 2019. Of the sixty-three eligible neonates, three parents declined consents, one died prior to the ROP examination, and nineteen were discharged prior to being enrolled. Hence, forty subjects were enrolled. Among these, two were discharged prior to being studied and five were unable to be studied due to contact isolation, hence thirty-three subjects had complete data for analysis; their characteristics are described in Table 1.

**Table 1:** Sample characteristics.

	Total sample (*N* = 33)				
	%	Mean	SD	Min	Max
Male sex	48	–	–	–	–
Birth weight (g)	–	1,117	338	490	1,970
PMA at exam (weeks)	–	33.5	2.1	30.3	38.4
Exam duration (mins)	–	3.7	1.9	2	11
Positive pressure respiratory support	52	–	–	–	–

PMA, postmenstrual age. Positive pressure respiratory support = high flow nasal cannula ≥2 L/min; CPAP, NIMV or SIMV; non-positive pressure respiratory support = low flow nasal cannula or room air.

### Statistical analysis

IBM-SPSS Windows® (version 26, USA) was used for statistical analyses. Descriptive statistics were calculated, and outliers and normality were checked for all variables prior to analysis. Because normality plots of distribution showed skewness for SC and HRV, non-parametric statistics were used for all analyses.

Friedman test was used to compare SC among baseline (pre-ROP examination), pain (ROP examination), and recovery (post-ROP examination) phases. In order to examine the contribution of HF-HRV pre-examination to infant stability (i.e., A/B/Ds) post-examination, linear regressions were used with natural log-transformed HF-HRV values to correct for skewness. For all analyses, *α* was set at <0.05 and two-tailed tests were used.

## Results

Seventy nine percent of the sample was White, non-Hispanic with a relatively equal distribution of male/female sex. Of the 33 subjects, 17 (52%) were on positive pressure respiratory support (i.e., high flow nasal cannula ≥2 L/min, CPAP, NIMV, SIMV), and 16 were on non-positive pressure respiratory support (i.e., low flow nasal cannula, room air) at the time of ROP examination (Table 1). Therefore, *post-hoc* analyses were conducted to examine the influence of respiratory support on SC, HF-HRV, and the association between HF-HRV and stability post-examination.

### Skin conductance (SC)

**Figure 2:**
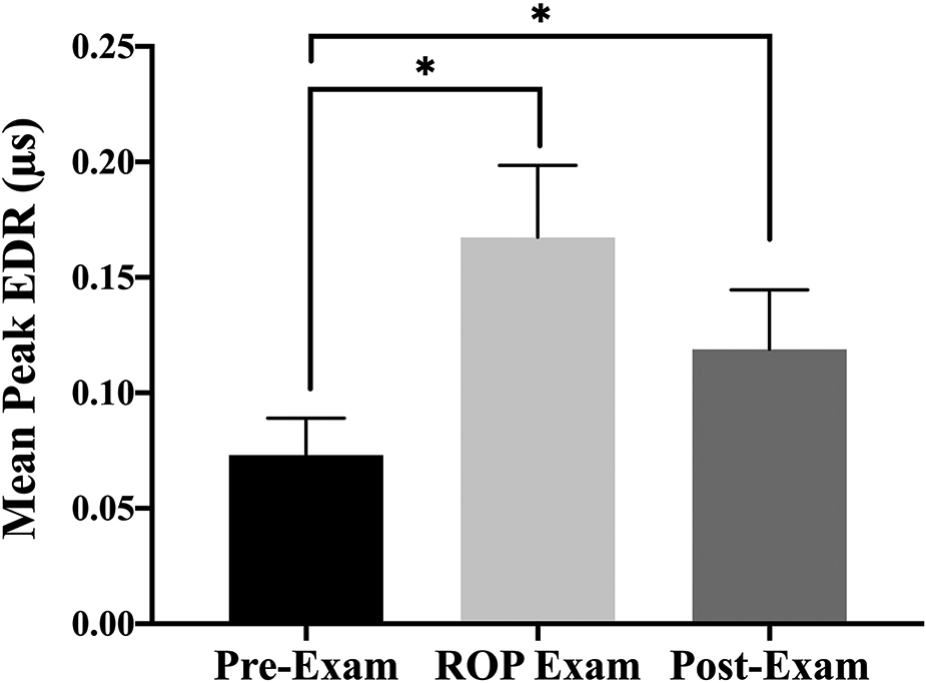
The bar graphs (SEM) illustrate mean of peaks (MPs) of skin conductance responses before the ROP examination, during the ROP examination, and after the ROP examination; *significant difference *p* < 0.0017 by Wilcoxon signed rank tests with Bonferroni correction applied.

The median, interquartile range (IQR) of SC responses were 0.37 (0.22–0.46), 0.35 (0.30–0.41), and 0.26 (0.15–0.39) EDRs/sec for pre-, during, and post-examinations, respectively. There were significantly lower EDRs/sec in the post-examination phase. In addition, there were statistically significant differences in SC mean of peaks (MPs) between phases, *χ*^2^(2) = 9.639, *p* = 0.008 (Friedman test). *Post hoc* analysis with Wilcoxon signed-rank tests was conducted with a Bonferroni correction applied, resulting in a significance level set at *p* < 0.017. Median (IQR) MPs for pre-, during, and post-examinations were 0.03 (0.02–0.08), 0.12 (0.03–0.24), and 0.05 (0.02–0.17), respectively. There was no significant difference between during and post-examinations (*Z* = −1.60, *p* = 0.11). Precisely, SC mean of peaks (MPs) during the exam phase was 56% higher than baseline (*Z* = −3.37, *p* < 0.001) and remained higher in the 20 min post-exam (*Z* = −2.42, *p* = 0.016) (Figure 2).

In order to examine the influence of positive vs. non-positive pressure respiratory support on SC, Mann-Whitney *U* tests were used for between-group comparisons. These comparisons showed no significant difference in SC response at any point between these two groups.

### Heart rate variability (HRV)

The median (IQR) of HF-HRV at 48- and 24 h prior to ROP examination were 3.67 (1.81–4.81) and 3.27 (1.36–5.28), respectively. There was no statistical difference in HF-HRV between these two time points. Infants on positive pressure respiratory support had median (IQR) HF-HRV at 48- and 24 h prior to ROP examination of 3.10 (0.84–4.19) and 1.54 (0.84–2.79), respectively. Infants on non-positive pressure respiratory support had median (IQR) HF-HRV at 48- and 24 h prior to ROP examination of 4.72 (3.36–7.36) and 4.19 (3.27–9.87), respectively. Group comparisons using Mann-Whitney *U* tests revealed that infants on positive pressure respiratory support had lower HF-HRV at both 48- and 24 h prior to the eye examination as compared with the non-positive pressure support group (*p* = 0.008 and *p* = <0.001, respectively).

### Infant stability post-ROP examination

During 72 h post-ROP examination, infants experienced on average 7 episodes of apneas, bradycardias, desaturations (A/B/Ds). For these A/B/Ds, infants required intervention in the range of mild to moderate stimulations [mean (SD) 1.8(2.0)]. Comparisons between the two respiratory support groups showed no difference in the frequency or required level of intervention for A/B/Ds during 72 h post-examination.

In order to examine the contribution of HF-HRV pre-examination to infant stability post-examination, linear regressions were used. No association was found between HF-HRV and frequency of A/B/Ds post-examination. However, significant models were found for HF-HRV at 48- and 24 h prior to examination and the level of intervention required for A/B/Ds post- examination [*F*(1, 30) = 6.62, *p* = 0.02 and *F*(1, 31) = 8.88, *p* = 0.01, respectively]. For HF-HRV at 48 h, it was found that the model explained 18% of the variance with HF-HRV significantly contributing to the level of intervention (*β* = −0.87, *p* = 0.02). The model with HF-HRV at 24 h explained 22% of the variance with HF-HRV significantly contributing to the level of intervention (*β* = −0.93, *p* = 0.01). These models indicated that infants with lower HF-HRV required higher level of intervention for A/B/Ds post-examination.

Considering the significant group difference in HF-HRV, the association between HF-HRV and level of intervention for A/B/Ds post-examination was probed by group. Linear regression showed significance only among infants on positive pressure support with the model explaining 25% of variance [*F*(1, 15) = 5.05, *p* = 0.04]. HF-HRV measured at 24 h prior to the eye-examination contributed to the level of intervention for A/B/Ds post-examination (*β* = −1.33, *p* = 0.04), indicating infants on positive pressure respiratory support with lower HF-HRV at 24 h pre-examination required higher level of intervention for A/B/Ds post-examination.

## Discussion

It is well established that ROP examinations including mydriatic administration, speculum insertion, and scleral depression cause pain and stress. Consequently, ROP examinations have been associated with adverse events including apneas, bradycardias, desaturations, elevated pain scores, and delayed gastric emptying (27–31). Despite evidence that ROP examinations are painful, and existence of standard criteria for ROP screening (3, 32–33), there is no consensus on pain relief for the ophthalmologic examination. The current use of analgesia including non-pharmacological (swaddling, oral sucrose, breastmilk, skin to skin care) and pharmacological (topical) analgesics is inconsistent and often suboptimal (29, 34–37). Recent studies have demonstrated the effectiveness of gentle human touch and intranasal fentanyl for pain management during ROP examinations underscoring optimization of pain control for these preterm infants (38, 39).

In addition to pain scales in common use with ROP examination, we found SC to provide an additional objective measure for assessing pain and recovery in preterm infants. SC is more sensitive than heart rate or blood pressure in infants (7, 15, 17, 40) because it is unaffected by hemodynamic changes (7, 20) and may be valuable with severely ill or very preterm/low-birthweight infants (16) who may be less robust in demonstrating distress behaviors (7). This subtlety of behavioral expression has potential for negatively impacting the accuracy of existing validated clinical pain scales (41) if used by novice observers who are inadequately trained to use the scale on preterm infants. In such scenarios, using SC as an adjuvant could be valuable for more accurate pain detection in this population.

Our findings for SC, specifically the amplitude of responses or mean of peaks (MPs) indicating strength of sympathetic firing, increased significantly with the ROP examination, and stayed elevated in the recovery phase compared to baseline. Previous studies showed similar findings when assessing infant SC response to painful procedures. Walas et al. (2021) measured preterm infants’ SC peaks per second using Med-Storm™ device 1 min prior to and 3 min after heel stick, and found a significant SC increase in pre- to post-heel stick (42). This increase was found regardless of the infants’ types of breathing (i.e., spontaneous or supported) (42). Similarly, Gendras et al. (2021) measured SC peaks per second from 2 min prior to through 2 min after painful, skin-breaking, or stressful (e.g., nursing care) event, and found SC to be a reliable measure of pain response (43). Moreover, the authors found peak pain responses to occur beyond 30 s post-procedure (43). Notably, we found significant rise in SC from baseline to eye examination with continued elevation through post-examination. This finding corroborates with previous study outcomes that infants’ heightened sympathetic activation persists even after the termination of painful or stressful event. This is also in support of studies that have shown elevated pain scores and physiologic disturbances up to 24 h after the ROP examinations [46–49]. Hence, we suggest that the pain and stress associated with ROP examinations are likely being underestimated.

In our study, we found that some infants appeared to be more adversely affected by the pain and stress from ROP examination than others. Nevertheless, no difference was found between the positive vs. non-positive pressure support groups on the frequency of or level of intervention needed for A/B/Ds post-ROP examination. This differs from findings in other studies where infants on oxygen support responded with more tachycardia to the mydriatic eye drops (cyclopentolate) and with subsequent bradycardia with the examinations compared to those on room air (27). The oxygen support group was found to have higher levels of circulating cyclopentolate compared to those in room air, although the same dose was administered (44). Importantly, we found that preterm infants on positive pressure support had lower HF-HRV, i.e., attenuated cardiac vagal tone, compared to the non-positive pressure support group. Moreover, lower HF-HRV contributed to higher intervention level for A/B/Ds post-ROP examination only among infants on positive pressure support. We speculate that lower resting parasympathetic tone places the individual at risk to repeated stress/pain exposures, and that this is the underlying characteristic associated with higher vulnerability to sequelae post-exam. Our findings of attenuated vagal tone with higher illness acuity and instability post-ROP exam for the higher acuity infants corroborates with other studies shown to use HRV to predict neonatal morbidity (24, 45, 46) and more specifically, HF-HRV to be associated with stress resiliency and to predict better health outcomes (24). Furthermore, this underscores that stress resilience is higher with vagal/sympathetic balance, i.e., vagal brake on sympathetic overdrive (46). Measurement of HF-HRV prior to the examination can be clinically useful in identifying infants more vulnerable to adverse events following stress/pain inducing procedures. With this identification, it may be cost effective to preemptively increase the infant's monitoring and respiratory support as applicable, while also ensuring adequate analgesia to prevent possible decompensation and care escalation.

In addition to potential prognostic value for vulnerability to adverse effects, HF-HRV has also been shown to be associated with severity of ROP disease where infants with more severe ROP requiring treatment were noted to have low HF-HRV compared to controls (2). Hence, perhaps, preterm infants should have their HF-HRV measured at baseline prior to the ROP screening examinations in order to anticipate their response to the examination as well as risk for disease.

The strengths of this study include its prospective nature; design controls including absence of a painful procedure within 12 h of ROP examination, consistency in care throughout the observation period, and that examinations were done by one pediatric ophthalmologist. Additionally, both SC and HRV are non-invasive measurements, reducing other confounding factors associated with invasive monitoring. Moreover, HRV was also standardized to control for confounders, i.e., circadian influence and behavioral state.

Limitations of the study include its small sample size and single center setting, hence limiting generalizability. This study, however, provides further compelling evidence on the stressful nature of the ROP examination causing sympathetic activation demonstrated by heightened amplitude of skin conductance. In addition, the findings provide insight into the underestimation of the adverse events associated with ROP examination and identification of infants who are more vulnerable to potential sequelae (i.e., events post-ROP examination). Our finding of the association between lower HF-HRV and the need for higher levels of A/B/D intervention, particularly among infants with positive pressure support, suggests the relevance of using HF-HRV as an index of stress/pain vulnerability to identify those individuals who are at higher risk for deterioration following invasive procedures in the NICU. We recommend deliberate discussions by the medical providers and nurses caring for these vulnerable infants to ensure adequate monitoring and analgesic care with ROP screening examinations as well as other painful procedures in the NICU.

## Data Availability

The raw data supporting the conclusions of this article will be made available by the authors, without undue reservation.
